# Supplementary material for: Spatio-temporal patterns of multi-trophic biodiversity and food-web characteristics uncovered across a river catchment using environmental DNA
Source: Commun Biol. 2022 Mar 23;5:259. doi: 10.1038/s42003-022-03216-z (PMC8943070; doi:10.1038/s42003-022-03216-z)
Supplement: Supplementary file 1 — Supplementary Information [file 42003_2022_3216_MOESM1_ESM.pdf]

Title: Spatio-temporal patterns of multi-trophic biodiversity and food-web characteristics uncovered across a river catchment using environmental DNA

Author list:

Rosetta C. Blackman[1, 2 *], Hsi-Cheng Ho[1], Jean-Claude Walser[3] and Florian Altermatt[1, 2 *]

Author affiliation:

[1] Eawag, Swiss Federal Institute of Aquatic Science and Technology, Department of Aquatic Ecology, Überlandstrasse 133, CH-8600 Dübendorf, Switzerland

[2] Department of Evolutionary Biology and Environmental Studies, University of Zurich, Winterthurerstr. 190, CH-8057 Zürich, Switzerland

[3] Genetic Diversity Centre (GDC), Department of Environmental Systems Sciences (DUSYS), Federal Institute of Technology (ETH), Rämistrasse 101, 8092 Zurich, Switzerland

*** Corresponding Authors:**

Email: rosiecblackman@gmail.com or florian.altermatt@eawag.ch

ORCID IDs:

Rosetta C. Blackman - https://orcid.org/0000-0002-6182-8691

Hsi-Cheng Ho - https://orcid.org/0000-0002-0734-0249

Jean-Claude Walser - https://orcid.org/0000-0003-1513-0783

Florian Altermatt- https://orcid.org/0000-0002-4831-6958

Supporting Information

Figures and Tables:

Figure S1: Drainage Area

Figure S2: Site genus richness from eDNA samples across all groups and Seasons

Figure S3: Food-web network with horizontal categories.

Figure S4: Supplementary food-web characteristics over space and time (Coherence, Number of links, Modularity, and Robustness)

Figure S5: Local food web structure: visualisation of the local food web at Site G_14

Table S1: MiSeq library loading and output information

Table S2: Alpha diversity from eDNA samples

Table S3: Function Feeding group categories

Table S4: Model comparison for mixed effect models with and without Drainage Area – Season interactions

Table S5: Mixed effect model outputs for all variables tested in this study - fixed effects.

Table S6: Mixed model outputs for all variables tested in this study - random effect.

Table S7: Fixed effect analysis of variants tables for all interaction models.

Table S8: Contrast testing output from emtrends(). The pairwise comparison of the mixed effect model slopes.

Table S9: Contrast testing output from emmeans(). The pairwise comparison of means between seasons for all variables included in the analysis.

Table S10: β-diversity against river distance for each group and each season.

Table S11: Primer selection for library preparation

Table S12: Positive sample information

Methods:

First PCR for library preparation (12S, COI and 16S)

Data preparation workflow - steps and parameters (12S, COI and 16S)

Figures and Tables:

Figure S1: Drainage Area: Drainage area per site sampled in this study.

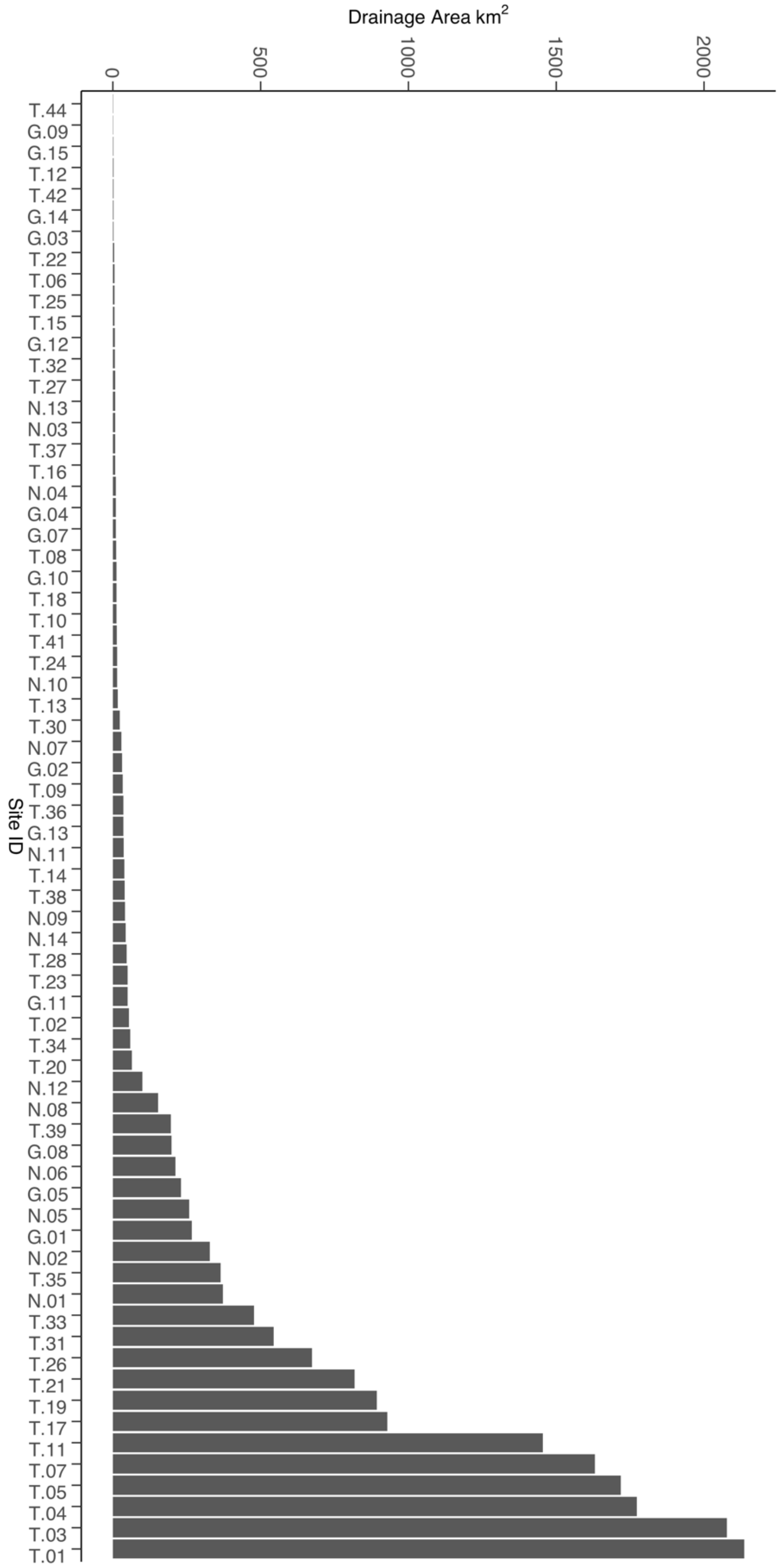

Figure S2: Site α-diversity (genus richness) from eDNA samples across all groups and Seasons: Bubble plot to show absolute number of genera recovered in each group from eDNA samples collected in Spring, Summer and Autumn.

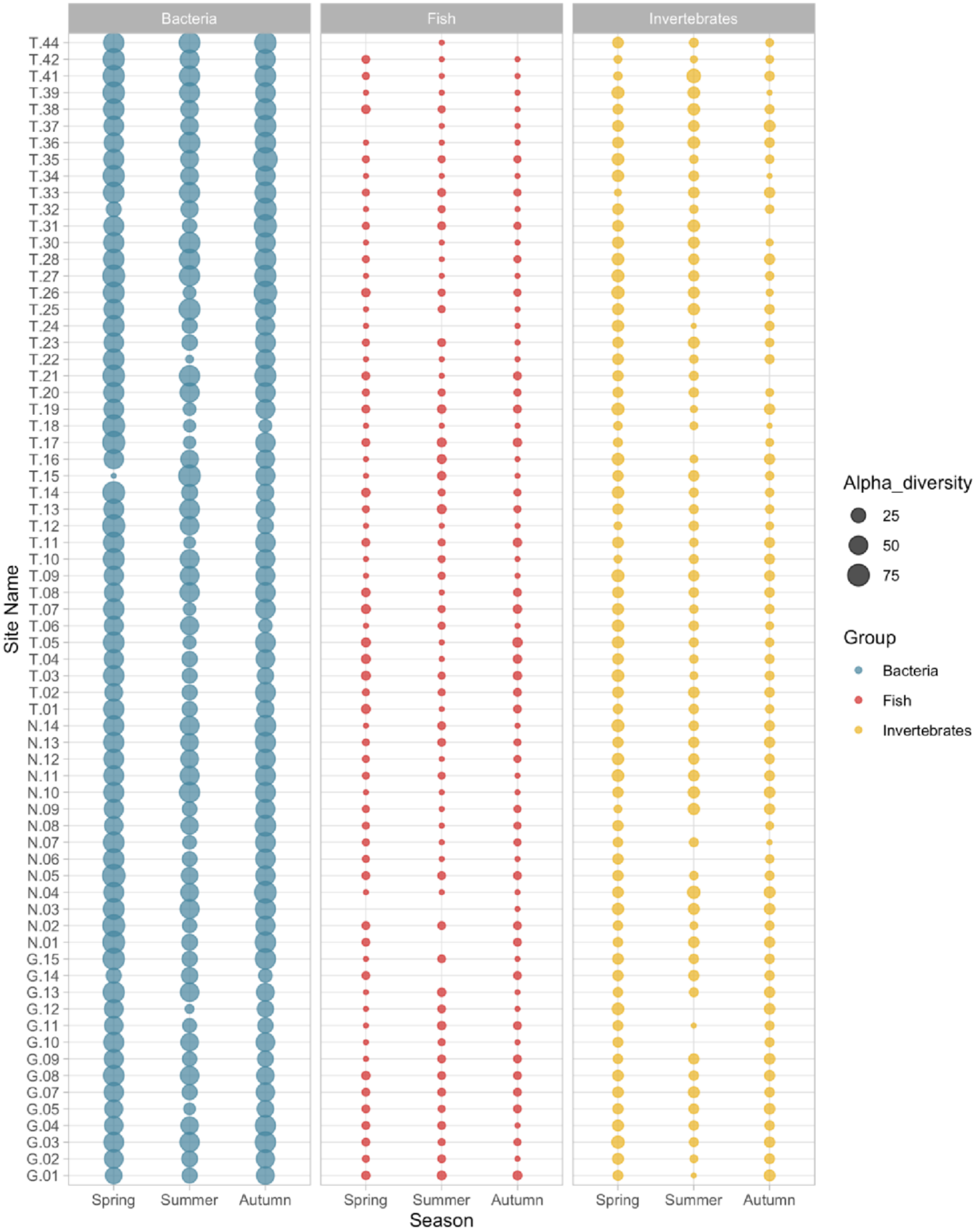

Figure S3: Food-web network with horizontal categories. This schematic shows the general groupings of the Functional Feeding Groups (FFG) present in our study: basal resource, decomposer, herbivore/detrivore and predator. This demonstrates the overlap some FFG may have in the overall food web. Colours and labels indicate the FFG.

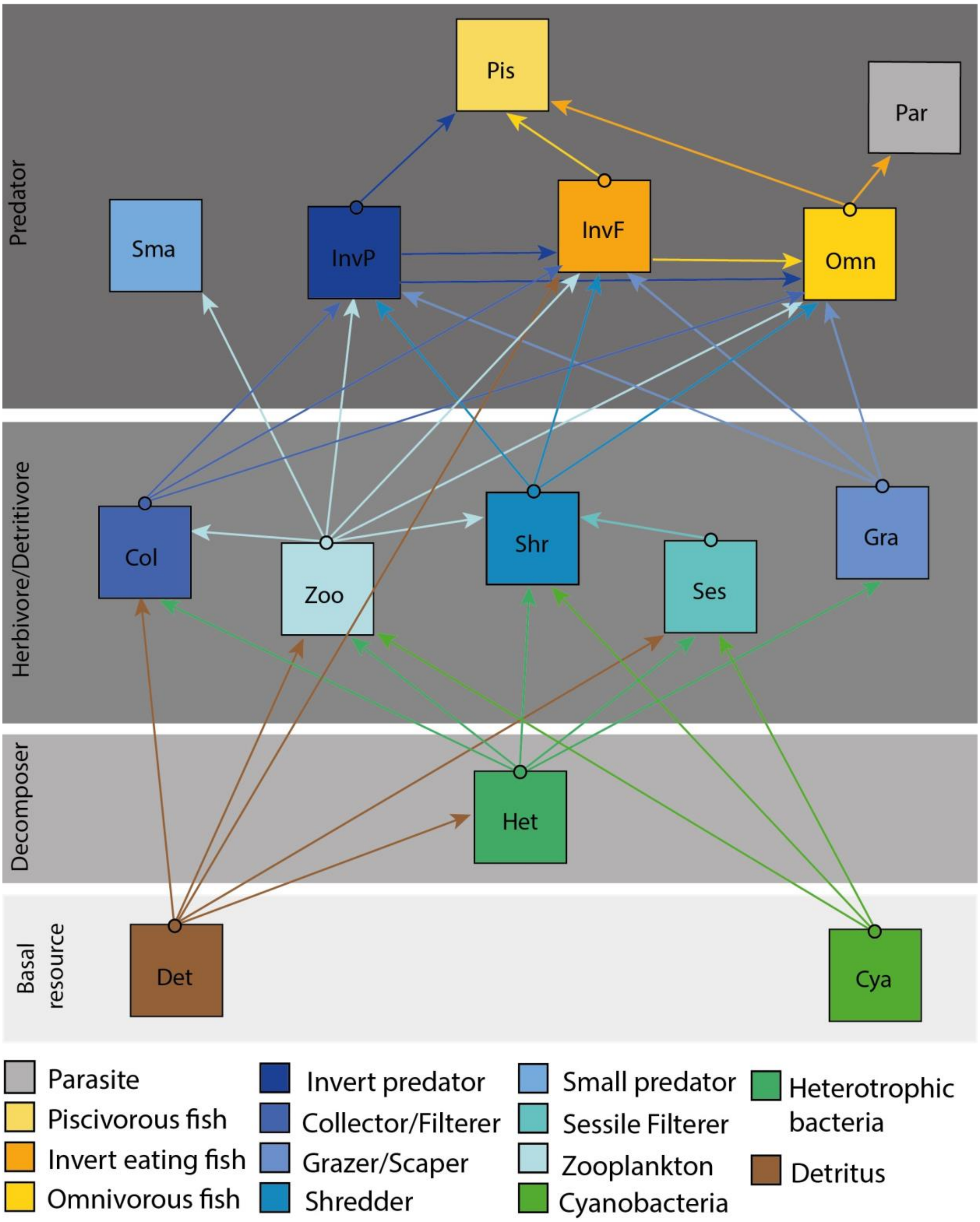

Figure S4: Supplementary food-web structural characteristics: Plots show foodweb structural characteristic: a and b: Coherence, c and d: Number of links, e and f: Modularity and g and h: Robustness. Plots a, c, e and g lines indicate linear mixed effect models with shaded area showing 95% confidence intervals as calculated using the model predictions and standard error. Plots a, c and e show the mixed effect models with a significant interaction with the difference across each season (colour represents season). Plot g shows no significant effect of the interaction between drainage area and season; therefore the blue line indicates the mixed effect model output of all the data as a function of drainage area in blue. Plots b, d, f and h show the change in characteristic over season with samples sites linked by grey lines over the three sampling seasons. Colour represents season: yellow – Spring, green – Summer and orange – Autumn.

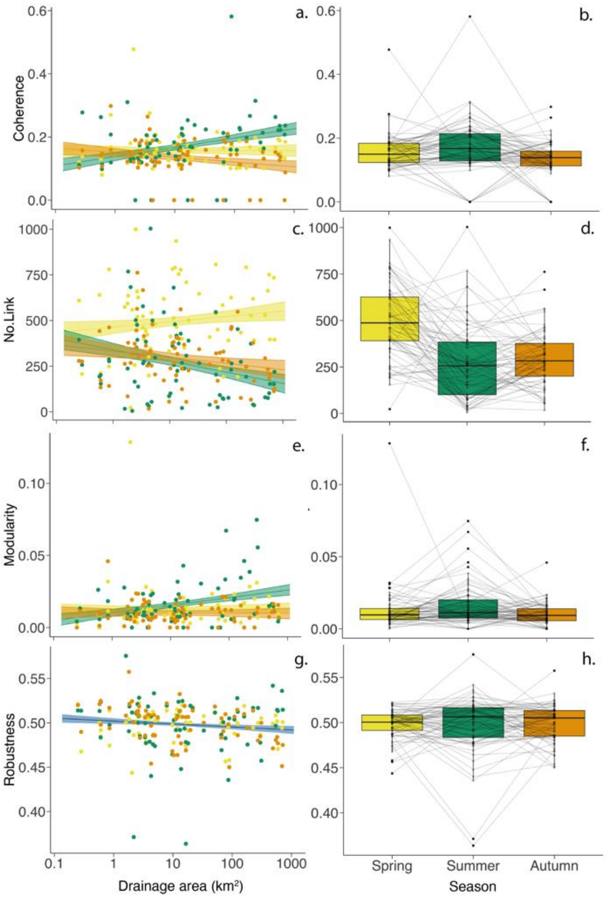

Figure S5: Local food web structure: visualisation of the local food web at Site G_14 across Spring, Summer and Autumn as generated using R package Cheddar.

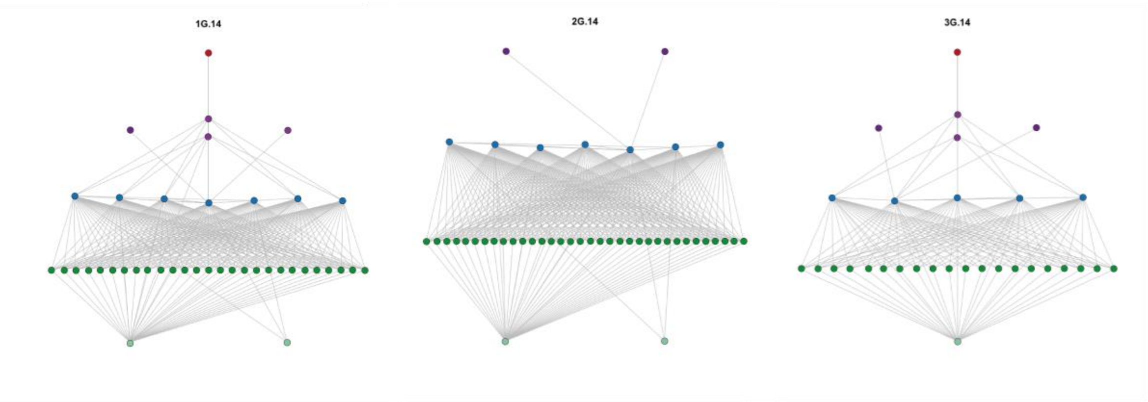

Table S1: MiSeq library loading, output and bioinformatics information.

	12S	COI	16S
**MiSeq regent kit**	250 cycles PE v2	600 cycle PE v3	600 cycle PE v3
**Target fragment**	278 bp	538 bp	450 bp
**library conc loaded**	17 pM	15 pM	15 pM
**PhiX conc loaded**	10 %	10 %	10 %
**Read lengths**	2x 125 cycles	2x 300 cycles	2x 300 cycles
**Number of raw reads**	12,291,563	14,325,071	14,027,834
**Q30**	95.8%	84.5%	77.5%
**After data processing**	10,880,390	12,777,525	12,563,934
**Average sequencing depth per sample**	43,176	50,704	49,857.
**OTUs (abundance threshold 2)**	371	13,009	34,102
**zOTUs (abundance threshold 7)**	252	6,685	30,788
**zOTUs with 97% clustering**	159	3,179	11,320

Table S2: Functional Feeding group categories: Descriptions used to establish interactives between genera of each group:

**Functional Feeding Group**	**Description**
Parasite	Feeds from host
Piscivorous fish	Feeds on fish
Invertebrate eating fish	Feeds on macroinvertebrates
Omnivorous fish	Feeds on both macroinvertebrate and detritus
Invertebrate predator	Macroinvertebrate which feeds on macroinvertebrates such as predatory Plecopterans
Collector/Filterer	Feeds on sedimented fine particle organic matter
Grazer/Scraper	Feeds on bacteria, biofilm, particulate organic matter, and living plants
Shredder	Feeds on fallen leaves, plant tissue and coarse organic matter
Small Predator	Small predators which feed on microscopic invertebrates and zooplankton
Sessile filterer	Non-mobile microscopic filter feeder which feed on bacteria and are grazed on by higher trophic levels (macroinvertebrate)
Zooplankton	The animal component of a planktonic community feeding on detritus, bacteria, biofilms and in some case other zooplankton
Heterotrophic bacteria	Bacteria which use organic carbon as a resource as opposed to autotrophs
Cyanobacteria	Basal resource, uses sunlight to generate resource
Detritus	This is the allochthonous inputs which consist of terrestrially derived carbon and forms the basal resource of aquatic food webs.

Table S3: Alpha diversity (Genus richness) from eDNA samples: reported for each group separately and combined and across each season and combined.

Group		Fish	Invertebrates	Bacteria	All genera
**Spring**	mean α-diversity	2.068	8.849	59.384	70
	standard deviation	1.316	2.970	12.572	12.582
	range	0-6	2-15	1-81	10-92
**Summer**	mean α-diversity	2.014	6.637	39.159	47.811
	standard deviation	1.254	3.808	16.787	18.339
	range	0-5	0-20	3-73	8-85
**Autumn**	mean α-diversity	1.849	5.410	55.520	62.780
	standard deviation	1.221	2.748	14.319	13.962
	range	0-6	0-10	17-90	19-96
**All seasons combined**	mean α-diversity	1.976	6.972	51.581	60.530
	standard deviation	1.77	3.490	16.95	17.668
	range	0-6	0-20	1-90	8-96

Table S4: Model comparison: mixed effect models with and without Drainage Area – Season interactions. P values < 0.05 indicate cases where the model with an interaction is the significantly better model.

	Model	npar	AIC	BIC	logLik	deviance	Chisq	Df	Pr(>Chisq)	
**Bacteria diversity**	No interaction	6	1702.4	1722.4	-845.2	1690.4				
	Interaction	8	1689	1715.7	-836.51	1673	17.389	2	0.0001675	***
**Fish diversity**	No interaction	6	635.97	655.97	-311.99	623.97				
	Interaction	8	616.37	643.03	-300.19	600.37	23.602	2	7.50E-06	***
**Invert diversity**	No interaction	6	1069.1	1089.1	-528.55	1057.1				
	Interaction	8	1070.6	1097.2	-527.29	1054.6	2.5147	2	0.2844	
**No. Link**	No interaction	6	2748.9	2768.9	-1368.4	2736.9				
	Interaction	8	2743.8	2770.4	-1363.9	2727.8	9.0981	2	0.01058	*
**Link density**	No interaction	6	908.82	928.82	-448.41	896.82				
	Interaction	8	908.74	935.4	-446.37	892.74	4.0792	2	0.1301	
**Nestedness**	No interaction	6	1169.2	1189.1	-578.57	1157.2				
	Interaction	8	1158.1	1184.8	-571.06	1142.1	15.036	2	0.0005433	***
**Coherence**	No interaction	6	-514.15	-494.16	263.08	-526.15				
	Interaction	8	-523.68	-497.02	269.84	-539.68	13.528	2	0.001154	**
**Connectance**	No interaction	6	-784.9	-764.9	398.45	-796.9				
	Interaction	8	-789.76	-763.09	402.88	-805.76	8.8558	2	0.01194	*
**Omnivory**	No interaction	6	-848.11	-828.11	430.05	-860.11				
	Interaction	8	-849.72	-823.06	432.86	-865.72	5.6119	2	0.06045	
**Robustness**	No interaction	6	-952.88	-932.88	482.44	-964.88				
	Interaction	8	-955.4	-928.74	485.7	-971.4	6.523	2	0.03833	*
**Modularity**	No interaction	6	-1194.7	-1174.7	603.33	-1206.7				
	Interaction	8	-1197	-1170.3	606.49	-1213	6.3267	2	0.04228	*
**F. diversity**	No interaction	6	669.07	689.06	-328.53	657.07				
	Interaction	8	670.93	697.59	-327.46	654.93	2.1392	2	0.3431	
**F. redundancy**	No interaction	6	957.6	977.59	-472.8	945.6				
	Interaction	8	948.25	974.92	-466.13	932.25	13.344	2	0.001266	**

106 **Table S5: Mixed effect model outputs for all variables tested in this study - fixed effects**
107 **outputs**

		Estimate	Std. Error	df	t value	Pr(>|t|)	
**Bacteria diversity**	(Intercept)	52.3401	2.7689	189.6787	18.903	< 2e-16	***
	DrainageAreaKmLog	0.9147	0.8219	189.6787	1.113	0.267149	
	SeasonSpring	3.4365	3.5615	134	0.965	0.336336	
	SeasonSummer	-5.6958	3.5615	134	-1.599	0.112123	
	DrainageAreaKmLog:SeasonSpring	0.2832	1.0571	134	0.268	0.789161	
	DrainageAreaKmLog:SeasonSummer	-3.7343	1.0571	134	-3.532	0.000565	***
**Fish diversity**	(Intercept)	0.95731	0.20884	184.78842	4.584	8.38E-06	***
	DrainageAreaKmLog	0.37095	0.06199	184.78842	5.984	1.11E-08	***
	SeasonSpring	0.08918	0.2626	134	0.34	0.73469	
	SeasonSummer	0.91455	0.2626	134	3.483	0.000671	***
	DrainageAreaKmLog:SeasonSpring	0.03738	0.07794	134	0.48	0.632311	
	DrainageAreaKmLog:SeasonSummer	-0.31722	0.07794	134	-4.07	7.99E-05	***
**Invert diversity**	(Intercept)	5.97672	0.61372	200.28743	9.739	< 2e-16	***
	DrainageAreaKmLog	-0.19869	0.18217	200.28743	-1.091	0.2767	
	SeasonSpring	2.54437	0.84943	134	2.995	0.00327	**
	SeasonSummer	1.12615	0.84943	134	1.326	0.18717	
	DrainageAreaKmLog:SeasonSpring	0.29175	0.25213	134	1.157	0.24927	
	DrainageAreaKmLog:SeasonSummer	-0.08574	0.25213	134	-0.34	0.73434	
**No. Link**	(Intercept)	333.057	35.52	186.621	9.377	< 2e-16	***
	DrainageAreaKmLog	-14.702	10.543	186.621	-1.394	0.16482	
	SeasonSpring	130.315	45.034	134	2.894	0.00445	**
	SeasonSummer	7.172	45.034	134	0.159	0.8737	
	DrainageAreaKmLog:SeasonSpring	27.387	13.367	134	2.049	0.04243	*
	DrainageAreaKmLog:SeasonSummer	-12.021	13.367	134	-0.899	0.37009	
**Link density**	(Intercept)	5.26487	0.41989	190.87454	12.539	< 2e-16	***
	DrainageAreaKmLog	-0.27555	0.12463	190.87454	-2.211	0.02823	*
	SeasonSpring	1.51821	0.54332	133.99999	2.794	0.00596	**
	SeasonSummer	0.22492	0.54332	133.99999	0.414	0.67955	
	DrainageAreaKmLog:SeasonSpring	0.30326	0.16127	133.99999	1.88	0.06222	.
	DrainageAreaKmLog:SeasonSummer	0.05447	0.16127	133.99999	0.338	0.73608	
**Nestedness**	(Intercept)	7.8394	0.7652	192.7018	10.245	< 2e-16	***
	DrainageAreaKmLog	-0.6424	0.2271	192.7018	-2.829	0.00517	**
	SeasonSpring	1.5652	0.9996	134	1.566	0.11974	
	SeasonSummer	-0.6552	0.9996	134	-0.655	0.51329	
	DrainageAreaKmLog:SeasonSpring	0.2129	0.2967	134	0.718	0.47419	
	DrainageAreaKmLog:SeasonSummer	1.0986	0.2967	134	3.703	0.00031	**
**Coherence**	(Intercept)	0.150699	0.013073	198.712332	11.528	< 2e-16	***
	DrainageAreaKmLog	-0.006515	0.00388	198.712332	-1.679	0.094713	.

	SeasonSpring	0.005908	0.017773	133.999994	0.332	0.740106	
	SeasonSummer	-0.012769	0.017773	133.999994	-0.718	0.473709	
	DrainageAreaKmLog:SeasonSpring	0.006584	0.005275	133.999994	1.248	0.214147	
	DrainageAreaKmLog:SeasonSummer	0.019278	0.005275	133.999994	3.654	0.000369	***
**Connectance**	(Intercept)	0.089993	0.006868	199.513706	13.103	< 2e-16	***
	DrainageAreaKmLog	-0.006097	0.002039	199.513706	-2.991	0.00313	**
	SeasonSpring	0.016533	0.009412	133.999991	1.757	0.08127	.
	SeasonSummer	0.007323	0.009412	133.999991	0.778	0.43791	
	DrainageAreaKmLog:SeasonSpring	0.003207	0.002794	133.999991	1.148	0.25299	
	DrainageAreaKmLog:SeasonSummer	0.008257	0.002794	133.999991	2.956	0.00369	**
**Omnivory**	(Intercept)	2.14E-02	5.93E-03	2.01E+02	3.604	0.000395	***
	DrainageAreaKmLog	-2.36E-03	1.76E-03	2.01E+02	-1.339	0.181926	
	SeasonSpring	9.45E-03	8.33E-03	1.34E+02	1.135	0.25838	
	SeasonSummer	4.60E-03	8.33E-03	1.34E+02	0.553	0.581217	
	DrainageAreaKmLog:SeasonSpring	9.42E-04	2.47E-03	1.34E+02	0.381	0.703515	
	DrainageAreaKmLog:SeasonSummer	5.45E-03	2.47E-03	1.34E+02	2.206	0.029077	*
**Func. Diversity**	(Intercept)	7.12634	0.23603	192.1954	30.193	<2e-16	***
	DrainageAreaKmLog	-0.11857	0.07006	192.1954	-1.692	0.0922	.
	SeasonSpring	0.55846	0.3075	134.00323	1.816	0.0716	.
	SeasonSummer	0.07407	0.3075	134.00323	0.241	0.81	
	DrainageAreaKmLog:SeasonSpring	0.0899	0.09127	134.00323	0.985	0.3264	
	DrainageAreaKmLog:SeasonSummer	-0.03882	0.09127	134.00323	-0.425	0.6713	
**Func. Redundancy**	(Intercept)	8.5479	0.4583	197.4572	18.653	< 2e-16	***
	DrainageAreaKmLog	0.3796	0.136	197.4572	2.791	0.005773	**
	SeasonSpring	0.3478	0.6166	134	0.564	0.573655	
	SeasonSummer	-0.7024	0.6166	134	-1.139	0.256683	
	DrainageAreaKmLog:SeasonSpring	-0.1492	0.183	134	-0.815	0.416435	
	DrainageAreaKmLog:SeasonSummer	-0.6448	0.183	134	-3.523	0.000584	***
**Robustness**	(Intercept)	0.509513	0.004668	188.093197	109.149	<2e-16	***
	DrainageAreaKmLog	-0.004027	0.001386	188.093197	-2.906	0.0041	**
	SeasonSpring	-0.010037	0.005959	134	-1.684	0.0944	.
	SeasonSummer	-0.012496	0.005959	134	-2.097	0.0379	*
	DrainageAreaKmLog:SeasonSpring	0.003614	0.001769	134	2.043	0.043	*
	DrainageAreaKmLog:SeasonSummer	0.004135	0.001769	134	2.338	0.0209	*
**Modularity**	(Intercept)	1.03E-02	2.57E-03	2.00E+02	4.026	8.06E-05	***
	DrainageAreaKmLog	-8.44E-05	7.62E-04	2.00E+02	-0.111	0.9119	
	SeasonSpring	2.22E-03	3.54E-03	1.34E+02	0.627	0.5317	
	SeasonSummer	-1.67E-04	3.54E-03	1.34E+02	-0.047	0.9624	
	DrainageAreaKmLog:SeasonSpring	1.91E-04	1.05E-03	1.34E+02	0.182	0.856	
	DrainageAreaKmLog:SeasonSummer	2.37E-03	1.05E-03	1.34E+02	2.257	0.0257	*

Table S6: Mixed model outputs for all variables tested in this study - Random effect

	Groups	Variance	Std.Dev.
**Bacteria diversity**	ID (Intercept)	34.65	5.887
	Residual	165.93	12.881
**Fish diversity**	ID (Intercept)	0.239	0.4889
	Residual	0.902	0.9498
**Invert diversity**	ID (Intercept)	0.4156	0.6447
	Residual	9.4386	3.0722
**No. Link**	ID (Intercept)	6479	80.49
	Residual	26529	162.88
**Link density**	ID (Intercept)	0.7512	0.8667
	Residual	3.8615	1.9651
**Nestedness**	ID (Intercept)	2.248	1.499
	Residual	13.07	3.615
**Coherence**	ID (Intercept)	0.0003392	0.01842
	Residual	0.004132	0.06428
**Connectance**	ID (Intercept)	7.53E-05	0.008679
	Residual	1.16E-03	0.034041
**Omnivory**	ID (Intercept)	1.41E-05	0.003752
	Residual	9.07E-04	0.030109
**F. diversity**	ID (Intercept)	0.2206	0.4696
	Residual	1.2369	1.1122
**F. redundancy**	ID (Intercept)	0.5204	0.7214
	Residual	4.9738	2.2302
**Robustness**	ID (Intercept)	0.0001056	0.01028
	Residual	0.0004645	0.02155
**Modularity**	ID (Intercept)	8.51E-06	0.002917
	Residual	1.64E-04	0.012801

Table S7: Fixed effect analysis of variants for all interaction models.

	Sum	Sq	Mean	Sq	NumDF	DenDF	Fvalue	Pr(>F)
**Bacteria diversity**	DrainageAreaKmLog	30.42	30.42	1	67	0.1834	0.6698821	
	Season	1113.21	556.61	2	134	3.3545	0.0378858	*
	DrainageAreaKmLog:Season	2985.99	1492.99	2	134	8.9977	0.0002155	***
**Fish diversity**	DrainageAreaKmLog	38.271	38.271	1	67	42.4266	1.11E-08	***
	Season	13.304	6.652	2	134	7.3745	0.0009153	***
	DrainageAreaKmLog:Season	22.546	11.273	2	134	12.497	1.06E-05	***
**Invert diversity**	DrainageAreaKmLog	13.304	13.304	1	67	1.4095	0.23933	
	Season	85.058	42.529	2	134	4.5059	0.01277	*
	DrainageAreaKmLog:Season	23.258	11.629	2	134	1.2321	0.29496	
**No. Link**	DrainageAreaKmLog	47193	47193	1	67	1.7789	0.1868	
	Season	280791	140396	2	134	5.2921	0.006136	**
	DrainageAreaKmLog:Season	242268	121134	2	134	4.5661	0.012068	*
**Link density**	DrainageAreaKmLog	13.744	13.7442	1	67	3.5593	0.06355	.
	Season	35.129	17.5644	2	134	4.5486	0.01227	*
	DrainageAreaKmLog:Season	15.523	7.7617	2	134	2.01	0.138	
**Nestedness**	DrainageAreaKmLog	24.758	24.758	1	67	1.8943	0.1733003	
	Season	68.101	34.05	2	134	2.6052	0.0776256	.
	DrainageAreaKmLog:Season	201.605	100.803	2	134	7.7125	0.0006755	***
**Coherence**	DrainageAreaKmLog	0.003169	0.0031692	1	67	0.767	0.384278	
	Season	0.004768	0.0023842	2	134	0.577	0.562956	
	DrainageAreaKmLog:Season	0.057028	0.0285141	2	134	6.9009	0.001404	**
**Connectance**	DrainageAreaKmLog	0.0038609	0.0038609	1	67	3.3319	0.07241	
	Season	0.003591	0.0017955	2	134	1.5495	0.21614	
	DrainageAreaKmLog:Season	0.0102912	0.0051456	2	134	4.4405	0.01357	*
**Omnivory**	DrainageAreaKmLog	0.000044	0.00004396	1	67	0.0485	0.82637	
	Season	0.0011682	0.0005841	2	134	0.6443	0.52664	
	DrainageAreaKmLog:Season	0.0050418	0.00252091	2	134	2.7808	0.06557	.
**Robustness**	DrainageAreaKmLog	0.001104	0.001104	1	67	2.3767	0.12787	
	Season	0.002293	0.0011465	2	134	2.4682	0.08858	.
	DrainageAreaKmLog:Season	0.0030127	0.0015064	2	134	3.243	0.04213	*
**Modularity**	DrainageAreaKmLog	0.00045633	0.00045633	1	67	2.7849	0.09982	.
	Season	0.00009284	0.00004642	2	134	0.2833	0.75374	
	DrainageAreaKmLog:Season	0.00103004	0.00051502	2	134	3.1432	0.04634	*
**F. Diversity**	DrainageAreaKmLog	5.9845	5.9845	1	66.999	4.8383	0.03129	*
	Season	4.8138	2.4069	2	134.003	1.9459	0.14687	
	DrainageAreaKmLog:Season	2.5894	1.2947	2	134.003	1.0467	0.35395	
**F. Redundancy**	DrainageAreaKmLog	8.961	8.961	1	67	1.8016	0.184056	
	Season	14.977	7.488	2	134	1.5055	0.225627	
	DrainageAreaKmLog:Season	67.664	33.832	2	134	6.8021	0.001536	**

Table S8: Contrast testing output from emtrends(). The pairwise comparison of the mixed effect model slopes

	contrast	estimate	SE	df	t.ratio	p.value	
**Bacteria diversity**	Spring - Summer	4.018	1.06	134	3.8	0.0006	***
	Spring - Autumn	0.283	1.06	134	0.268	0.9612	
	Summer - Autumn	-3.734	1.06	134	-3.532	0.0016	**
**Fish diversity**	Spring - Summer	0.3546	0.0779	134	4.549	<.0001	****
	Spring - Autumn	0.0374	0.0779	134	0.48	0.8811	
	Summer - Autumn	-0.3172	0.0779	134	-4.07	0.0002	***
**Invert diversity**	Spring - Summer	0.3775	0.252	134	1.497	0.2955	
	Spring - Autumn	0.2918	0.252	134	1.157	0.4809	
	Summer - Autumn	-0.0857	0.252	134	-0.34	0.9383	
**No. Link**	Spring - Summer	39.4	13.4	134	2.948	0.0105	
	Spring - Autumn	27.4	13.4	134	2.049	0.1047	
	Summer - Autumn	-12	13.4	134	-0.899	0.6417	
**Link density**	Spring - Summer	0.2488	0.161	134	1.543	0.2744	
	Spring - Autumn	0.3033	0.161	134	1.88	0.1483	
	Summer - Autumn	0.0545	0.161	134	0.338	0.9391	
**Nestedness**	Spring - Summer	-0.886	0.297	134	-2.985	0.0094	**
	Spring - Autumn	0.213	0.297	134	0.718	0.7535	
	Summer - Autumn	1.099	0.297	134	3.703	0.0009	***
**Coherence**	Spring - Summer	-0.01269	0.00528	134	-2.406	0.0457	**
	Spring - Autumn	0.00658	0.00528	134	1.248	0.4271	
	Summer - Autumn	0.01928	0.00528	134	3.654	0.0011	**
**Connectance**	Spring - Summer	-0.00505	0.00279	134	-1.808	0.171	
	Spring - Autumn	0.00321	0.00279	134	1.148	0.4864	
	Summer - Autumn	0.00826	0.00279	134	2.956	0.0102	**
**Omnivory**	Spring - Summer	-0.004509	0.00247	134	-1.825	0.1654	
	Spring - Autumn	0.000942	0.00247	134	0.381	0.923	
	Summer - Autumn	0.005451	0.00247	134	2.206	0.0738	
**Func. Diversity**	Spring - Summer	0.1287	0.0913	134	1.41	0.3385	
	Spring - Autumn	0.0899	0.0913	134	0.985	0.5876	
	Summer - Autumn	-0.0388	0.0913	134	-0.425	0.9052	
**Func. Redundancy**	Spring - Summer	0.496	0.183	134	2.708	0.0208	**
	Spring - Autumn	-0.149	0.183	134	-0.815	0.6943	
	Summer - Autumn	-0.645	0.183	134	-3.523	0.0017	**
**Robustness**	Spring - Summer	-0.000521	0.00177	134	-0.295	0.9533	
	Spring - Autumn	0.003614	0.00177	134	2.043	0.1059	
	Summer - Autumn	0.004135	0.00177	134	2.338	0.054	
**Modularity**	Spring - Summer	-0.002179	0.00105	134	-2.075	0.099	
	Spring - Autumn	0.000191	0.00105	134	0.182	0.9819	
	Summer - Autumn	0.002371	0.00105	134	2.257	0.0656	

Table S9: Contrast testing output from emmeans(). The pairwise comparison of means between seasons for all variables included in the analysis

	contrast	estimate	SE	df	t.ratio	p.value	
**Bacteria diversity**	Spring - Summer	19.8	2.19	134	9.027	<.0001	****
	Spring - Autumn	4.19	2.19	134	1.91	0.1398	
	Summer - Autumn	-15.61	2.19	134	-7.117	<.0001	****
**Fish diversity**	Spring - Summer	0.1159	0.162	134	0.717	0.7539	
	Spring - Autumn	0.1884	0.162	134	1.165	0.476	
	Summer - Autumn	0.0725	0.162	134	0.448	0.8953	
**Invert diversity**	Spring - Summer	2.42	0.523	134	4.627	<.0001	****
	Spring - Autumn	3.319	0.523	134	6.345	<.0001	****
	Summer - Autumn	0.899	0.523	134	1.718	0.2023	
**No. Link**	Spring - Summer	227.8	27.7	134	8.213	<.0001	****
	Spring - Autumn	203	27.7	134	7.321	<.0001	****
	Summer - Autumn	-24.7	27.7	134	-0.892	0.6462	
**Link density**	Spring - Summer	1.95	0.335	134	5.84	<.0001	****
	Spring - Autumn	2.32	0.335	134	6.944	<.0001	****
	Summer - Autumn	0.37	0.335	134	1.104	0.513	
**Nestedness**	Spring - Summer	-0.131	0.615	134	-0.212	0.9754	
	Spring - Autumn	2.13	0.615	134	3.461	0.0021	**
	Summer - Autumn	2.261	0.615	134	3.674	0.001	***
**Coherence**	Spring - Summer	-0.015	0.0109	134	-1.372	0.3583	
	Spring - Autumn	0.0234	0.0109	134	2.137	0.0863	
	Summer - Autumn	0.0384	0.0109	134	3.509	0.0018	***
**Connectance**	Spring - Summer	-0.0042	0.0058	134	-0.724	0.7498	
	Spring - Autumn	0.025	0.0058	134	4.322	0.0001	***
	Summer - Autumn	0.0292	0.0058	134	5.046	<.0001	****
**Omnivory**	Spring - Summer	-0.00712	0.00513	134	-1.39	0.3492	
	Spring - Autumn	0.01195	0.00513	134	2.331	0.0549	
	Summer - Autumn	0.01907	0.00513	134	3.721	0.0008	***
**Func. Diversity**	Spring - Summer	0.826	0.189	134	4.363	0.0001	***
	Spring - Autumn	0.797	0.189	134	4.21	0.0001	***
	Summer - Autumn	-0.029	0.189	134	-0.153	0.9872	
**Func. Redundancy**	Spring - Summer	2.3658	0.38	134	6.231	<.0001	****
	Spring - Autumn	-0.0482	0.38	134	-0.127	0.9911	
	Summer - Autumn	-2.414	0.38	134	-6.358	<.0001	****
**Robustness**	Spring - Summer	0.001075	0.00367	134	0.293	0.9538	
	Spring - Autumn	-0.000443	0.00367	134	-0.121	0.992	
	Summer - Autumn	-0.001518	0.00367	134	-0.414	0.9101	
**Modularity**	Spring - Summer	-0.0034	0.00218	134	-1.56	0.2667	
	Spring - Autumn	0.00273	0.00218	134	1.251	0.4255	
	Summer - Autumn	0.00613	0.00218	134	2.811	0.0156	*

Table S10: β-diversity against river distance for each group and each season. β-diversity is calculated based on Jaccard dissimilarity where 0 would indicate identical communities. β diversity was further separated into genus loss (Nestedness) and replacement (Turnover). Correlations reported are based on Mantel statistic and p-values (significant p-values are highlighted in bold)

Group	Season	Jaccard dissimilarity	Taxon loss (Nestedness)	Taxon replacement (Turnover)
Fish	Spring	0.1434<br>**p = 0.002**	0.2187<br>**p = 0.001**	0.07043<br>p = 0.891
	Summer	0.1712<br>**p = 0.001**	-0.08374<br>p = 0.989	0.2031<br>**p = 0.001**
	Autumn	0.3214<br>**p = 0.001**	0.0587<br>**p = 0.05**	0.2691<br>**p = 0.001**
Invertebrate	Spring	0.0952<br>**p = 0.031**	-0.00215<br>p = 0.464	0.06638<br>p = 0.051
	Summer	0.1685<br>**p = 0.001**	-0.07856<br>p = 0.976	0.142<br>**p = 0.002**
	Autumn	0.1114<br>**p = 0.013**	-0.01447<br>p = 0.597	0.07066<br>**p = 0.047**
Bacteria	Spring	-0.05822<br>p = 0.834	-0.0655<br>p = 0.928	0.01599<br>p = 0.34
	Summer	**0.1785**<br>**p = 0.003**	0.08859<br>**p = 0.044**	0.06969<br>p = 0.068
	Autumn	0.06779<br>p = 0.12	-0.04308<br>p = 0.747	0.1185<br>**p = 0.014**

Table S11: Primer selection for library preparation: 12S and COI primers included a modification to include the Nextera® transposase sequences in the first PCR, whereas 16S included this modification in the second PCR.

Marker	Fragment	Forward primer	Reverse primer	Reference
**12S**	**106 bp**	5’-TACTGGGATTAGATACCCC-3’	5’-CTAGAACAGGCTCCTCTAG-3	[14]
**COI**	**313 bp**	mICOIintF: 5’-GGWACWGGWTGAACWGTWTAYCCYCC-3’	jgHCO2198: 5’-TAIACYTCIGGRTGICCRAARAAYCA-3’	[15, 16]
**16S**	**450 bp**	U341F: 5’-CCTACGGGDGGCWGCA-3’	U806R: 5’GACTACHVGGGTMTCTAATC-3’	[17]

Table S12: Positive sample information. For each library a positive control was included to be used in determining possible contamination within the library preparation.

Marker	Type	Sequence if applicable
**12S**	**Tissue extraction (*Gadus morhua*)**	TACNTNNTGTTATGGTTCCGTTTAACATTGATG<br>GTTTTATTACCCAAACCATNCCGCCTGGGAAC<br>TACGAGCAATAGCTTAAAACCCAAAGGACTTN<br>GGCGGTGCTTTAGACCCCCCTAGAGGAGCCTG<br>TTCTAGCTGTCTCTTATACACATCTCCGGCCCA<br>CGAGACNGAGCTAGAACNAGCTCCTCTNGNGG<br>GTNTAANGCCCGCCANTCCTTTGGGTTTTAAG<br>CTATTGCTCGTANNTCCAGCGATGGTTTGGGTA<br>TAAAACATCAATGTTAACNACCATACTNNTGG<br>GGTATCTAATCCAGTACTGTCTCTTATACCGTC<br>TACGCTCCNCANCCCTGTTAGACGCATATTGAA<br>TGNGATCGCCGGTCGGGGTATGAAAAACAACG<br>GCANCNCCGGNCAANACATCACACCCGCCNGG<br>AAAAACCAAACCNGCNTTTNTAAACCTGCTTGC<br>ACAGGCCGTNCGCGTCATTCTTCACGGNGNCGC<br>ATGTTTTNGTTTCGNCATTTTGCCGCCTGCGTGG<br>GCTGCGTGAGCAGCACACCCAGCCAGCCCCTTN<br>NCTGCCGTTGNCGGNGNCAANNGCNTCNGGACA<br>GCACTCCNGCNTGNCTCGTCTAGCACCACCACA<br>CNACAGCANAGAGCNGATGCTCNCCGCCAACGA<br>AGCCAGGNNGNTGNTTGAGATNCNTAAGTATNA<br>CCTACTGGCGCNNNATCTTGAGATNCGCGTCCA<br>AGTCTAGCCNATAATNNGCAAGATAGNTTGTGT<br>CNAGGCNGTCATCGACNTCAGCAACGCTGNGNT<br>TANNGNTNGTTG
**COI**	**Synthetic sequence**	GGAACAGGTTGAACTGTATATCCCCCATCAACC<br>TAGTTACGAAGAGCTATAGATCATATAATCCTT<br>AAGTGGAATGTTAATGTGAGTTCAATATGATAC<br>ACGCCACAGACTCATGTATGTGGATCGGAAGCC<br>AGCTGTTTCCGACCTCGGAGCCGAGAGTGGTTT<br>CTGAATTACACATGTAAGATAAAATCATTAAAG<br>GTACTAACTCACGAAACCTCAGGATATGCGTGG<br>TTTGCTGAGATTTCTATTTTCTCGTTCTTGATTTA<br>ACCACGTAAAATGTGTGAAAACTAAAGGTTCTA<br>GCATTTCTAAGGATCACTACGCCTAACGTCTCAC<br>TTTATCTTAAATTTGATTTTTTGGTCACCCTGAAGT<br>TTA
**16S**	**Bacteria stock community**	*Bacillus subtilis*, *Bacillus brevis* and *Serratia fonticuli*

Methods:

First PCR for library preparation

12S - Vertebrate library

The first PCR was carried out in a total volume of 25 µL containing: 0.5 µM each primer, 0.4 mg/mL BSA (New England Biolabs, Ipswich, MA, USA), 12.5 µL Q5® HighFidelity 2X Master Mix (New England Biolabs), and 2 µL of DNA template per reaction. PCR profiles were as follows: initial denaturation 98 °C for 5 min followed by 35 cycles of 98 °C for 10 s, 58 °C for 20 s, and 72 °C for 30 s, and a final extension step of 72 °C for 7 min, before the plates were cooled down to 10 °C.

COI - Metazoan library

The first PCR was carried out in a total volume of 25 µL containing: 0.5 µM each of each primer, AmpliTaq Gold 360° (1.25 U/µL), 1x Buffer I (Thermo Fisher Scientific, MD, USA), 0.1 mg/µL BSA, 0.2 mM dNTP, 1 mM $MgCl_2$, SigmaFree water and 2 µL of DNA template per reaction. PCR profiles were carried out using a touchdown protocol as follows: initial denaturation at 95 °C for 10 min, the first 25 cycles started with the denaturation at 95 °C for 15 s, annealing at 62 °C for 30 s, followed by extension at 72 °C for 30 s. After this the cycler performed 16 cycles where the annealing temperature was reduced by one degree each cycle, performing the last cycle at a temperature of 45 degrees. Final extension was performed at 72 °C for 5 min before the plates were cooled down to 10 °C.

16S – Archaea and Bacteria library

Library preparation for the 16S V3-V4 region followed a three-step PCR library preparation using the method described in [18] with minor modifications. In brief, the first PCR was performed in 15 µL volumes containing: 0.5 µM of each forward and reverse primer, 1x supplied buffer (Faststart TAQ, Roche, Inc., Basel, Switzerland), 1 mg/µL BSA, 0.18 mM dNTPs, 2.0 mM $MgCl_2$, 0.05 units per µL Taq DNA polymerase (Faststart TAQ, Roche, Inc.) and 2 µL of DNA template per reaction. This was carried out in

triplicate and then pooled. Each plate was then cleaned using Exo I Nuclease (EXO I) and Shrimp Alkaline Phosphatase (SAP) (Thermo Fisher Scientific Inc., Waltham, Maryland USA). The master mix consisted of 1.6 U/µL Exo I and 0.15 U/µL SAP in a total volume of 1.1 µL which was then added to 7.5 µL of the PCR product. Products were heated to 37 °C for 15 minutes, followed by 15minutes at 80 °C, and was then cooled to 4 °C and stored at –20 °C. The second PCR was conducted with the same PCR conditions as the first PCR except the forward and reverse primers were modified to include the Nextera® transposase sequences (Microsynth, AG, Balgach, Switzerland) and only 1 µL of cleaned PCR product was used in the reaction.

All cleaned amplicons (12S, COI and 16S) were the indexed using unique combinations of the Illumina Nextera XT Index Kit (see Methods).

Data preparation workflow - steps and parameters:

12S

Step A - Data Quality Check: usearch v11.0.667

Step B - Trimming and Merging: usearch v11.0.667

Trim: R1:30nt, R2:40nt

Merge: Min. overlap: 15 bp, Max difference: 10, Min Identity (%): 70, Min. Merged length: 50 and Min. merged quality: 0.

Step C - Trim Full-Length Primer Sites: usearch v11.0.667

Amplicon range: 100-300

No. of mismatches: 1

Coverage: full-length

Step D: Size selection and quality filtering: PRINSEQ-lite 0.20.4

Size Range: 100-250

GC Range: 30-70

Min Q Mean: 20

Step E - Clustering - UNOISE for ZOTUs: usearch v11.0.667

Min Abundance Size: 10

Step F - Taxonomic Assignment: SINTAXv11.0.667

Confidence threshold: 0.85

Reference: NCBI BLAST[19] based reference (v200416)

COI (Metazoan)

Step A - Data Quality Check: usearch v11.0.667

Step B - Trimming and Merging: usearch v11.0.667

Trim: R1: 40nt, R2: 50nt

Merge: Min. overlap: 40 bp, Min Identity (%): 70, Min. Merged length: 100

Step C - Trim Full-Length Primer Sites: usearch v11.0.667

Amplicon range: 100-600

No. of mismatches: 1

Coverage: full-length

Step D: Size selection and quality filtering: PRINSEQ-lite 0.20.4

Size Range: 300-450

GC Range: 30-70

Min Q Mean: 20

Step E - Clustering - UNOISE for ZOTUs: usearch v11.0.667

Step F - Taxonomic Assignment Predictions: SINTAX v11.0.667

Confidence threshold: 0.85

Reference: Custom made reference database consisting of MIDORI[20] (v20180221) and EPT (v200420). Supplemented with unpublished macroinvertebrate sequences.

16S (Archaea and Bacteria)

Step A - Data Quality Check: usearch v11.0.667

Step B - Trimming and Merging: usearch v11.0.667 and FLASH v1.2.11

Trim: R1: 20nt, R2: 50nt

Merge: Min. overlap: 15 bp, Max. overlap: 300

Step C - Trim Full-Length Primer Sites: usearch v11.0.667

Amplicon range: 100-2000

No. of mismatches: 1

Coverage: full-length

Step D: Size selection and quality filtering: PRINSEQ-lite 0.20.4

Size Range: 300-500

GC Range: 30-70

Min Q Mean: 20

Low complexity filter: dust (30)

Step E - Clustering – UNOISE3 for ZOTUs: usearch v11.0.667

Step F - Taxonomic Assignment Predictions: SINTAX v11.0.667

Confidence threshold: 0.85

References: SILVA[21] (V128)

Supplementary References

The references below with * were used to create the Functional Feeding Group information see Supplementary Data File 1. The information was gathered from Schmidt-Kloiber, A and Hering, D. (2015) www.freshwaterecology.info – an online tool that unifies standardises and codifies more than 20,000 European freshwater organisms and their ecological preferences. Ecological Indicators, 53: 271-282. Accessed on the 1st October 2020

1. Newton, R. J., Jones, S. E., Eiler, A., McMahon, K. D. & Bertilsson, S. A guide to the natural history of freshwater lake bacteria. *Microbiol. Mol. Biol. Rev.* **75**, 14–49 (2011)*
2. Tierno de Figueroa, J. M. & López-Rodríguez, J. M. Trophic ecology of Plecoptera Insecta: a review. *Eur. Zool J.* **861,** 79-102 (2019) *
3. Car, M. *et al.* Diptera except Chironomidae authors depending on family. in *Fauna Aquatica Austriaca* (ed. Moog, O.) Water Management Cadastre 543 (Federal Ministry of Agriculture, Forestry, Environment and Water Management, Vienna, 2002) *
4. Eder, E. *et al.* Crustacea authors depending on taxagroup. in *Fauna Aquatica Austriaca* (ed. Moog, O.) Water Management Cadastre 543 (Federal Ministry of Agriculture, Forestry, Environment and Water Management, Vienna, 2002) *
5. Bauernfeind, E., Moog, O. & Weichselbaumer, P. Ephemeroptera. in *Fauna Aquatica Austriaca* (ed. Moog, O.) Water Management Cadastre 543 (Federal Ministry of Agriculture, Forestry, Environment and Water Management, Vienna, 2002) *
6. Grenouillet, G. & Schmidt-Kloiber, A. Fish Indicator Database. Eurolimpacs project, Workpackage 7 - Indicators of ecosystem health, Task 4 (2006) Accessed via www.freshwaterecology.info, version 7.0 accessed on 08.10.2020.*
7. Merritt, R. W., Cummins, K. W., & Berg, M. (eds.). An introduction to the aquatic insects of North America. (Iowa: Kendall/Hunt Publishing Company, 2008).
8. Graf, W., Grasser, U. & Weinzierl, A. Plecoptera. in *Fauna Aquatica Austriaca* (ed.

Moog, O.) Water Management Cadastre (Federal Ministry of Agriculture, Forestry, Environment and Water Management, Vienna, 2002) *

9. Hörner, K., Moog, O. & Sporka, F. Oligochaeta. in *Fauna Aquatica Austriaca* (ed. Moog, O.) Water Management Cadastre 543 (Federal Ministry of Agriculture, Forestry, Environment and Water Management, Vienna, 2002) *
10. Janecek, B. F. U., Moog, O., Moritz, C., Orendt, C. & Saxl, R. Chironomidae authors depending on subfamily. in *Fauna Aquatica Austriaca* (ed. Moog, O.) Water Management Cadastre 543 (Federal Ministry of Agriculture, Forestry, Environment and Water Management, Vienna, 2002) *
11. Schmedtje, U. & Colling, M. Ecological typing of the aquatic macrofauna. Information reports of the Bavarian State Office for Water Management 4/96, 543 pp. (1996) *
12. Wöss, E. Bryozoa. in *Fauna Aquatica Austriaca* (ed. Moog, O.) Water Management Cadastre 543 (Federal Ministry of Agriculture, Forestry, Environment and Water Management, Vienna, 2002) *
13. Nesemann, H. & Reischütz, P. L. Gastropoda. in *Fauna Aquatica Austriaca* (ed. Moog, O.) Water Management Cadastre 543 (Federal Ministry of Agriculture, Forestry, Environment and Water Management, Vienna, 2002) *
14. Kelly, R. P., Port, J. A., Yamahara, K. M. & Crowder, L. B. Using environmental DNA to census marine fishes in a large mesocosm. *PLoS One* **9**, e86175 (2014).
15. Leray, M. *et al.* A new versatile primer set targeting a short fragment of the mitochondrial COI region for metabarcoding metazoan diversity: application for characterizing coral reef fish gut contents. *Front. Zool.* **10**, 34 (2013).
16. Geller, J., Meyer, C., Parker, M. & Hawk, H. Redesign of PCR primers for mitochondrial cytochrome c oxidase subunit I for marine invertebrates and application in all-taxa biotic surveys. *Mol. Ecol. Resour.* **13**, 851–861 (2013).

17. Liu, C. M. *et al*. BactQuant: An enhanced broad-coverage bacterial quantitative real-time PCR assay. BMC Microbiology 12, 56 (2012)
18. Mansfeldt, C. *et al.* Microbial community shifts in streams receiving treated wastewater effluent. *Sci. Total Environ.* **709**, 135727 (2020).
19. National Center for Biotechnology Information (NCBI). Bethesda (MD): National Library of Medicine (US), National Center for Biotechnology Information; [1988] – Available from: https://www.ncbi.nlm.nih.gov/ [accessed 2020 April 16th].
20. Leray, M., Ho, S-L., Lin, I-J. & Machida, R.J. MIDORI server: a webserver for taxonomica assignment of unknown metazoan mitochondrial-encoded sequences using cueate database. *Bioinformatics*, 34: 21, 3753-3754 (2018).
21. Quast. C., *et al.* The SILVA ribosomal RNA gene database project: improved data processing and web-based tools. *Nucl. Acids Res.* 41: 590-596 (2013).
