# Supplementary material for: Spatio-temporal patterns of multi-trophic biodiversity and food-web characteristics uncovered across a river catchment using environmental DNA
Source: Commun Biol. 2022 Mar 23;5:259. doi: 10.1038/s42003-022-03216-z (PMC8943070; doi:10.1038/s42003-022-03216-z)
Supplement: Supplementary file 2 — Description of Additional Supplementary Files [file 42003_2022_3216_MOESM2_ESM.pdf]

Description of Additional Supplementary File

File name: Supplementary Data File 1

Description: Food web classification.

All aquatic associated genus were classified into Functional Feeding groups to construct the metaweb used for food web analysis. This table has the details of which groups each genus was classified into, a general descriptor of that group and the information source used to derive the groupings.
