# Supplementary material for: Spatio-temporal patterns of multi-trophic biodiversity and food-web characteristics uncovered across a river catchment using environmental DNA
Source: Commun Biol. 2022 Mar 23;5:259. doi: 10.1038/s42003-022-03216-z (PMC8943070; doi:10.1038/s42003-022-03216-z)
Supplement: Supplementary file 4 — Reporting Summary [file 42003_2022_3216_MOESM4_ESM.pdf]

nature portfolio

Corresponding author(s): Rosetta Blackman

Last updated by author(s): 3rd February 2022

Reporting Summary

Nature Portfolio wishes to improve the reproducibility of the work that we publish. This form provides structure for consistency and transparency in reporting. For further information on Nature Portfolio policies, see our Editorial Policies and the Editorial Policy Checklist.

Statistics

For all statistical analyses, confirm that the following items are present in the figure legend, table legend, main text, or Methods section.

n/a	Confirmed	
☐	☒	The exact sample size (*n*) for each experimental group/condition, given as a discrete number and unit of measurement
☐	☒	A statement on whether measurements were taken from distinct samples or whether the same sample was measured repeatedly
☐	☒	The statistical test(s) used AND whether they are one- or two-sided<br>*Only common tests should be described solely by name; describe more complex techniques in the Methods section.*
☐	☒	A description of all covariates tested
☐	☒	A description of any assumptions or corrections, such as tests of normality and adjustment for multiple comparisons
☐	☒	A full description of the statistical parameters including central tendency (e.g. means) or other basic estimates (e.g. regression coefficient) AND variation (e.g. standard deviation) or associated estimates of uncertainty (e.g. confidence intervals)
☐	☒	For null hypothesis testing, the test statistic (e.g. *F*, *t*, *r*) with confidence intervals, effect sizes, degrees of freedom and *P* value noted<br>*Give P values as exact values whenever suitable.*
☒	☐	For Bayesian analysis, information on the choice of priors and Markov chain Monte Carlo settings
☒	☐	For hierarchical and complex designs, identification of the appropriate level for tests and full reporting of outcomes
☒	☐	Estimates of effect sizes (e.g. Cohen's *d*, Pearson's *r*), indicating how they were calculated

Our web collection on statistics for biologists contains articles on many of the points above.

Software and code

Policy information about availability of computer code

Data collection	Details of all field sampling, sample processing and eDNA sequencing is detailed in the manuscript
Data analysis	All data analysis was carried out in R version 4.0.4.

For manuscripts utilizing custom algorithms or software that are central to the research but not yet described in published literature, software must be made available to editors and reviewers. We strongly encourage code deposition in a community repository (e.g. GitHub). See the Nature Portfolio guidelines for submitting code & software for further information.

Data

Policy information about availability of data

All manuscripts must include a data availability statement. This statement should provide the following information, where applicable:

- Accession codes, unique identifiers, or web links for publicly available datasets
- A description of any restrictions on data availability
- For clinical datasets or third party data, please ensure that the statement adheres to our policy

Sequencing data generated during this study is available on the European Nucleotide Archive accession numbers XXXXXXX – XXXXXXXX. The data and analysis files used in this study are available on the following GitHub repository: https://github.com/RosettaBlackman/RiverDNA

Field-specific reporting

Please select the one below that is the best fit for your research. If you are not sure, read the appropriate sections before making your selection.

☐ Life sciences ☐ Behavioural & social sciences ☒ Ecological, evolutionary & environmental sciences

For a reference copy of the document with all sections, see nature.com/documents/nr-reporting-summary-flat.pdf

Ecological, evolutionary & environmental sciences study design

All studies must disclose on these points even when the disclosure is negative.

Study description	This study looked at the biodiversity of fish, invertebrates and bacteria across a spatio-temporal field study in switzerland. Using environmental DNA we constructed biodiversity patterns and food web dynamics
Research sample	Metabarcoding data from 282 Genera from Fish, Macroinvertebrate and Bacteria groups
Sampling strategy	Environmental DNA, 2x 500ml of water
Data collection	All eDNA samples were extracted and then sequenced using primer pairs which target 12S, COI and 16S. These libraries were then sequenced on the MiSeq and subsequently taxonomically assigned to relevant reference databases
Timing and spatial scale	Samples were collected in May, August and October 2018.<br>Sequencing took place in January - June 2019<br>Analysis thereafter
Data exclusions	Non-aquatic taxa were excluded from the data analysis
Reproducibility	The DNA is still available from these samples and the sequencing can be repeated.<br>All analysis has been can be repeated, all raw and assigned data files for this project are present in the GitHub repository
Randomization	Samples were not collected in random, i.e., samples were collected starting at the downstream part of the catchment working upstream. This so as to not collect any eDNA which may have been dispersed by disturbance of sample upstream
Blinding	Samples were extracted at random.

Did the study involve field work? ☒ Yes ☐ No

Field work, collection and transport

Field conditions	eDNA samples were always collected a minimum of two days after rainfall. The 73 field sites were samples across 4 days maximum.
Location	River Thur catchment, Switzerland
Access & import/export	All sites are publically accessible
Disturbance	The collection of eDNA requires minimal habitat disturbance and samplers did not enter the watercourse to collect samples.

Reporting for specific materials, systems and methods

We require information from authors about some types of materials, experimental systems and methods used in many studies. Here, indicate whether each material, system or method listed is relevant to your study. If you are not sure if a list item applies to your research, read the appropriate section before selecting a response.

Materials & experimental systems

n/a	Involved in the study	
☒	☐	Antibodies
☒	☐	Eukaryotic cell lines
☒	☐	Palaeontology and archaeology
☒	☐	Animals and other organisms
☒	☐	Human research participants
☒	☐	Clinical data
☒	☐	Dual use research of concern

Methods

n/a	Involved in the study	
☒	☐	ChIP-seq
☒	☐	Flow cytometry
☒	☐	MRI-based neuroimaging
